# A multi-site, year-round turbulence microstructure atlas for the deep perialpine Lake Garda

**DOI:** 10.1038/s41597-021-00965-0

**Published:** 2021-07-22

**Authors:** Sebastiano Piccolroaz, Bieito Fernández-Castro, Marco Toffolon, Henk A. Dijkstra

**Affiliations:** 1 Ecole Polytechnique Fédérale de Lausanne, Physics of Aquatic Systems Laboratory, Margaretha Kamprad Chair, School of Architecture, Civil and Environmental Engineering, Lausanne, CH-1015 Switzerland; 2 Utrecht University,Institute for Marine and Atmospheric research Utrecht, Department of Physics, Utrecht, 3584 CC the Netherlands; 3 University of Southampton, Ocean and Earth Science, National Oceanography Centre, Southampton, SO14 3ZH UK; 4 University of Trento, Department of Civil, Environmental and Mechanical Engineering, Trento, I-38123 Italy

**Keywords:** Limnology, Physical oceanography

## Abstract

A multi-site, year-round dataset comprising a total of 606 high-resolution turbulence microstructure profiles of shear and temperature gradient in the upper 100 m depth is made available for Lake Garda (Italy). Concurrent meteorological data were measured from the fieldwork boat at the location of the turbulence measurements. During the fieldwork campaign (March 2017-June 2018), four different sites were sampled on a monthly basis, following a standardized protocol in terms of time-of-day and locations of the measurements. Additional monitoring activity included a 24-h campaign and sampling at other sites. Turbulence quantities were estimated, quality-checked, and merged with water quality and meteorological data to produce a unique turbulence atlas for a lake. The dataset is open to a wide range of possible applications, including research on the variability of turbulent mixing across seasons and sites (demersal vs pelagic zones) and driven by different factors (lake-valley breezes vs buoyancy-driven convection), validation of hydrodynamic lake models, as well as technical studies on the use of shear and temperature microstructure sensors.

## Abstract (summary)

**Table:** 

Measurement(s)	temperature of water • conductivity of water • pressure of water • velocity shear • turbidity • concentration of chlorophyll a in water • turbulence related quantities (TKE and temperature variance dissipation rates, diapycnal diffusivity) • Secchi depth • temperature of air • atmospheric pressure • atmospheric wind speed • humidity • solar radiation
Technology Type(s)	turbulence microstructure profiler • Secchi disk • weather station
Sample Characteristic - Environment	lake
Sample Characteristic - Location	Italy • Lago di Garda

Machine-accessible metadata file describing the reported data: 10.6084/m9.figshare.14564430

## Background & Summary

Natural water bodies such as lakes are turbulent, density-stratified environments. As such, vertical exchanges of momentum, heat and mass are dominated by turbulence, which acts to stir the fluid increasing irreversible fluxes at the molecular scale. In this way, turbulent fluxes control a large plethora of physical, ecological, and chemical processes including the supply of nutrients into the photic zone allowing plankton photosynthesis^1^, the ventilation of oxygen-depleted benthic layers^2^, and the exchange of gases with the atmosphere^3^.

Direct measurement of turbulent fluxes is difficult to accomplish in natural systems^4^. For this reason, indirect methods are typically used, as those based on the use of microstructure profilers^5,6^. Microscale observations allow to quantify bulk turbulence-related quantities such as dissipation rates of turbulent kinetic energy (TKE) *ε* and of temperature variance *χ*. Then, these quantities are used to estimate turbulent diffusivity according to widely employed models based on the TKE^7^ and temperature variance^8^ conservation equations. Although microstructure measurements have the considerable advantage of directly observing turbulence, the *in-situ* operation of microstructure profilers is a time and labor intensive activity, which historically hampered the systematic acquisition of temporally and spatially resolved turbulence data. Here we challenge this operational burden by making available one of the few comprehensive turbulence dataset existing for a lake.

Between March 2017 and June 2018, an extensive turbulence monitoring campaign was carried out in Lake Garda (Italy) using a microstucture profiler. Along with turbulence related quantities, CTD (conductivity and temperature) and water quality (chlorophyll-a and turbidity) profiles, and meteorological data were measured. All vertical profiles were measured down to 100 m depth, while meteorological data were collected directly at the fieldwork boat, providing representative weather conditions at the time and location of the turbulence measurements. The monitoring campaign was performed on a regular monthly basis, with occasional activities added to the routine schedule.

The microstructure measurements and estimated turbulence-related quantities were quality-controlled and then merged with meteorological data to produce a comprehensive dataset for Lake Garda. This dataset comprises the first systematic turbulence measurements acquired for this lake (before that, only circumscribed turbulence profiles were measured^9^) and complements high-resolution measurements of deep-interior mixing acquired between May 2017 and May 2018 by a moored station at the lake’s deepest point^10,11^. Besides the synchronous collection of unprecedented measurements for Lake Garda, the present dataset is one of the few extensive turbulence datasets available for a lake and, to the best of our knowledge, the first accessible in a public repository. Microstructure data are available for some lakes, but their resolution is often limited in space and time to a particular location^12–15^ and/or to a particular time of the year^16–19^, or focus on the characterization of specific processes^20–24^.

A unique feature of the dataset is its year-round and multi-site nature. This allows to explore the heterogeneity of turbulence and mixing processes across seasons and sites and, together with concurrent water quality and open-lake meteorological measurements, contributes to improve our knowledge of Lake Garda. However, the relevance of the dataset is broad and not limited to research focused on this lake (please, see the ‘Usage Notes’ section). Considering the general scarcity of turbulence measurements, this dataset provides useful information to improve our understanding of turbulent mixing in deep perialpine lakes in general, of which Lake Garda is a meaningful example. Likewise, the combined availability of shear and fast-response thermistors data can contribute to define improved post-processing strategies to broaden the applicability of these sensors. In this regard, here we provide a step-by-step description of the processing approach used to analyze the data, trying to condensate into a self-contained document the best practices available in the literature.

We highlight that the current attention of the scientific community towards this type of measurements is high, as evidenced by, e.g., the ongoing “Léman exploration (LéXPLORE)” project (https://lexplore.info/)^25^, aimed at regularly collecting high-resolution data from an interdisciplinary floating laboratory on Lake Geneva, and the “ATOMIX” working group of the Scientific Committee on Oceanic Research (https://scor-int.org/) aimed at developing best practices for acquiring, processing, and sharing turbulence observations. The topic is therefore timely and cutting-edge to the field of limnology.

## Methods

### Instruments

#### Turbulence microstructure and water quality profiler

High-resolution turbulence profiles were collected using a free-falling, internally recording microstructure profiler MicroCTD, specifically developed by Rockland Scientific International Inc. (RSI) for application in lakes, reservoirs and estuaries.

The instrument has a length of 1 m, a maximum operational depth of 100 m and is equipped with turbulence and water quality sensors located at the front bulkhead, which is protected by a sensor guard. Turbulence properties were measured with two microstructure airfoil shear probes and two fast-response temperature sensors (type FP07), sampled at high frequency (512 Hz) and having accuracy and resolution of: <5% of measured value and 10^−3^ s^−1^ for velocity shear, and <0.005 °C and 10^−5^ °C for temperature, respectively. The two shear probes were positioned orthogonal to each other to measure both components of the horizontal velocity shear fluctuations $\left(\frac{{\rm{\partial }}{u}^{{\rm{{\prime} }}}}{{\rm{\partial }}z},\,,{\rm{a}}{\rm{n}}{\rm{d}},\,,\frac{{\rm{\partial }}{v}^{{\rm{{\prime} }}}}{{\rm{\partial }}z}\right)$. Hereafter, the ′ denotes turbulent quantities, i.e., the fluctuation relative to the average value. The vertical positioning of the profiler is recorded with a pressure sensor, sampling at 64 Hz and with an accuracy and resolution of 0.1 bar (i.e., 0.1% of full scale) and 5 × 10^−4^ bar, respectively. Water quality profiles were measured with a stable and reliable conductivity/temperature (CT) sensor and a fluorescence/turbidity (FT) sensor (JFE-Advantech Sensors) having accuracy and resolution of: <0.01 °C and 0.001 °C for temperature, <0.01 mS cm^−1^ and 0.001 mS cm^−1^ for conductivity, <4 ppb (i.e., <1% of full scale) and 0.01 ppb for fluorescence, and <0.3 FTU or <2% of measured value and 0.03 FTU for turbidity. The sampling rate is 64 Hz for the CT sensor and 512 Hz for the FT sensor. Finally, a two-axis vibration sensor (i.e., a pair of piezo-accelerometers) sampling at 512 Hz and a two-axis inclinometer (pitch and roll angles accurate to 0.1°) sampling at 64 Hz monitored the dynamics of the profiler flight.

The instrument’s buoyancy was regulated to achieve a downward profiling speed (*W*) of about 0.75 m s^−1^ (specifically, *W* = 0.74 ± 0.04 m s^−1^, mean ± standard deviation, based on the entire dataset). This profiling speed is within the range recommended for turbulence measurements using shear probes: sufficiently high to satisfy Taylor’s frozen turbulence hypothesis^26^ (i.e., the measurement interval is short compared to the time scale of evolution of turbulence properties) and to achieve an appropriate angle of attack of shear probes^27^, and low enough to adequately resolve the higher wavenumbers. The resolution of small-scale fluctuations is limited by the finite size of the shear probes. To account for this, the measured signal was corrected for the limited spatial response^27^. The resolution of temperature fluctuations is instead limited by the time response of the thermistors (∼5–10 ms). For this reason, analogous instruments equipped with fast-response thermistors are typically operated at lower profiling speed in the order of 0.1 m s^−1^ (below the required range for the shear probes), in order to allow to better resolve the higher wavenumbers of temperature gradient spectra^12^. Here we challenged the fast-response thermistors with a higher profiling speed than typically used, trying to find a compromise to exploit the capabilities of the two types of sensors across the wide range of turbulence intensities observed in the lake.

#### Meteorological station

Meteorological ground stations operated by local organizations (e.g., local Environmental Protection Agencies - EPAs) are available along the perimeter of the lake. However, atmospheric conditions at the lake region are complex and also strongly affected by small-scale factors due to local topography^28,29^. Indeed, the wind speed above the lake was reported to be on average 1.6 times more intense than the one measured by a nearby ground station^30^. In order to rely on meteorological data that were representative of the open-lake conditions, the fieldwork boat was equipped with a weather station throughout most of the monitoring campaign (Davis Vantage Pro2 6152). The station included temperature, humidity, wind speed, atmospheric pressure, and solar radiation sensors. The station was operating between September 14th, 2017 and May 8th, 2018. Before September 14th, 2017 the fieldwork boat was equipped with a thermohygrometer. The thermohygrometer and the weather station were operated together during an overlapping period, allowing to cross validate the temperature and humidity measurements acquired by the two instruments. The weather sensors were installed at about 2 m above lake surface.

### Monitoring sites and protocol

**Fig. 1:**
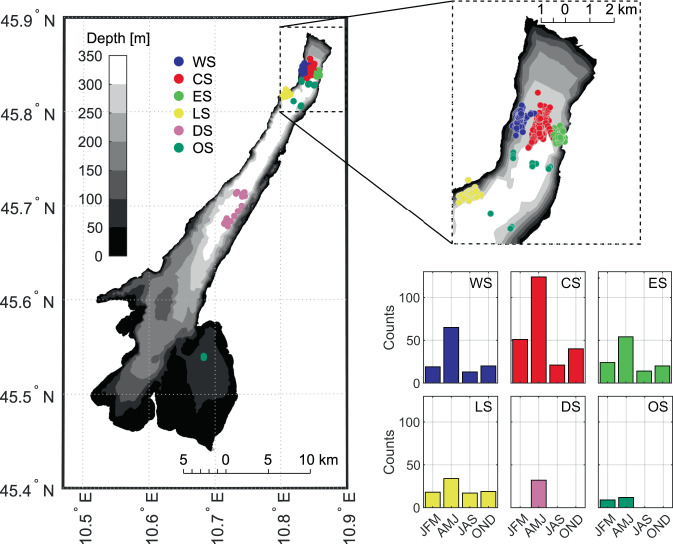
Spatial and temporal distribution of the microstructure measurements. Bathymetry of Lake Garda and location of measured vertical profiles grouped by monitoring site (different colors). The seasonal distribution of the measured profiles is also shown.

Four reference sampling sites were established in the northern, deep and elongated part of Lake Garda: three along a transverse transect where the lake is about 2.5 km wide (West Station - WS, Central Station - CS, and East Station - ES), and one in a sheltered bay a few kilometers (_~_4 km) to the south (Limone Station - LS). The bathymetric map of the lake with the location of the monitoring sites is shown in Fig. 1. The four sites were sampled on a monthly basis, following a standard procedure aimed at minimizing the differences among the monitoring days in terms of scheduling of the fieldwork activity (time-of-day and monitoring sites sequence). Figure 1 also shows the time distribution of the vertical profiles grouped by monitoring site and season. Specifically, the monitoring activity was carried out between about 10:00 and 16:00 local time and (mostly) with the following sequence: CS, ES, WS, LS, and back to CS. We note that CS was sampled twice per day, in the morning and in early afternoon, for the majority of the monitoring days. This allowed acquiring turbulence and water quality profiles in two time-of-day characterized by different wind patterns, according to the regular lake-valley breezes that develop at the lake especially during sunny and warm summer days. The northerly Peler breeze blows during the night and in the morning, and the southerly Ora del Garda breeze blows late in the morning and in the afternoon^31^. To achieve good statistical significance, a minimum of three and up to six vertical profiles were measured at each reference site.

The monitoring campaign included also other activities, such as an intensive 24-hour session in May 2018 (May 7th - 8th), and the occasional collection of vertical profiles at additional locations. These sites included the deepest point of the lake (Deep Station - DS) and a shallower site in the south-east basin, besides occasional profiles in other points of the lake (Other Stations - OS). Overall, 606 profiles were measured.

The fieldwork activity was conducted from an inflatable rubber motor boat. A Secchi disk (black and white, 20 cm diameter) completed the fieldwork boat’s on-board equipment, and the anecdotal experience of the boat captain enriched the fieldwork activity.

### Processing of microstructure data

The processing of the microstructure data was based on the scripts provided by RSI (ODAS libraries v4.4), properly modified for the specific purposes of the analysis. The data processing workflow is described in detail below, aiming at providing a self-contained, step-by-step reference compendium synthesizing the key procedures and the best practices available in the literature.

The signal produced by shear probes and fast-response thermistors were converted into physical units knowing the sensitivity of the shear probes (provided with the calibration certificate) and the resistance of the FP07, respectively. The coefficients of Steinhart-Hart equation describing the variation of the FP07’s resistance with temperature were calibrated independently for each cast using the precise CT sensor as a reference. Specifically, a second-order Steinhart-Hart polynomial was used if the observed temperature range was sufficiently wide (larger than 8°C, see Technical Note 5 available at https://rocklandscientific.com in the Support section) while its linear form was used for narrower temperature ranges. The vertical profile was partitioned into 3 m long, 50% overlapping segments. For each segment, the frequency (*f* ) spectra of shear ${\widetilde{\Psi }}_{S}(f)$ and temperature gradient ${\widetilde{\Psi }}_{T}(f)$ were derived by ensemble averaging the fast Fourier transform power spectra computed for 1 m long, 50% overlapping subsegments, detrended and Hanning tapered. With an acquisition frequency of 512 Hz and a profiling speed *W* ~ 0.75 m s^−1^, each 1 m long subsegment was formed by about 700 datapoints.

The measured frequency spectrum $\widetilde{\Psi }(f)$ was converted into the corresponding wavenumber spectrum Ψ(*k*) knowing the profiling speed *W* and according to the Taylor’s frozen turbulence hypothesis^26^, which allows relating time and length scales as follows

1 $$\Psi (k)=W\widetilde{\Psi }(f),\quad {\rm{and}}\quad k=f/W.$$

We note that here all wavenumbers are defined cyclicly, that is with units of cycle per meter (cpm). Multiplying by the factor 2*π* provides the corresponding circular wavenumber expressed in radians per meter. Before calculating the shear spectra Ψ_*S*_, the raw microstructure shear signals were cleaned by removing spikes (typically due to collision with particles such as phytoplankton) and by high-pass filtering with a cut-off wavenumber corresponding to half of the instrument length, i.e., *k* = 1/0.5 cpm^32^ to remove the noise originating from the low-frequency motions of the free-falling profiler. Each shear spectrum was corrected accounting for the probe’s spatial response according to a first-order low-pass filter with a half-power wavenumber of 48 cpm following ref. ^27^, i.e., by dividing the shear spectra by the transfer function

2 $$H(k)=\frac{1}{1+{\left(k/48\right)}^{2}}.$$

Contamination by higher-frequency instrument vibrations were corrected using the piezo-accelerometers data and following the Goodman method^33^ for removal of coherent noise from the measured shear spectra.

The temperature gradient spectra Ψ_*T*_ were derived from the vertical temperature gradient calculated by applying a high-pass filter to the pre-emphasized temperature signal^34^. The spectra were corrected for the frequency response of the probes by taking into account a sensor-dependent time constant *τ*. This time constant is related to the thermal transfer rate across the glass coating and the boundary layer around the sensor. The time response of individual fast thermistors is poorly known, and it is sensor dependent since they are “handmade” and thus have slight physical differences^35^. Furthermore, previous research has shown that the effective time constant *τ* depends on the thickness of the diffusive layer around the sensor tip, which is related to the profiling speed. Hence, the effective time response decreases with the profiling speed according to a velocity scaling of the form $\tau ={\tau }_{0}{\left(\frac{W}{{W}_{0}}\right)}^{\gamma }$, where *W*_0_ = 1 m s^−1^, *γ* is a negative exponent and the sensor-specific time response *τ*_0_ = *τ* at *W* = *W*_0_ (ref. ^36^). Besides the fact that *τ*_0_ is an *a-priori* unknown sensor-dependent constant, the appropriate value for *γ* and whether a single- or double-pole correction should be adopted, are also not well established^35–39^.

In order to implement an appropriate time response correction, we performed a trial-and-error sensitivity analysis and found that the best match between *ε* estimates based on FP07 and shear probes (which were taken as a reference) was achieved when correcting the observed temperature gradient spectra using a single pole transfer function

3 $$H(f)=\frac{1}{1+{\left(f/{f}_{c}\right)}^{2}},$$

where the cut-off frequency of the filter ${f}_{c}=\frac{1}{2\pi \tau }$, *γ* = −0.12 and *τ*_0_ = 7 × 10^−3^ s. These values were coherent with previous findings^38^, and confirm the weak dependence on the profiling speed *W* reported previously^40^, and the value of *τ*_0_ is consistent with the nominal response time of FP07 sensors. Given the satisfactory results achieved (see the ‘Technical Validation’ section) and the agreement with previous literature values, we used this setup for the analysis while referring more systematic correction and uncertainty analyses to future dedicated studies (see also the ‘Usage Notes’ section).

The de-spiked and response-corrected shear and temperature gradient wavenumber spectra, processed as outlined above, were used to calculate vertical profiles of dissipation rates of turbulent kinetic energy *ε* and temperature variance *χ*, according to the procedure described in the next section. Henceforth, Ψ, *ε*, and *χ* written with subscripts *S* and *T* refer to quantities evaluated based on shear and temperature microstructure measurements, respectively, while when written without subscripts they keep their general meaning.

### Estimation of *ε* and *χ*

Under the assumption of small-scale isotropy, *ε* can be evaluated from one single shear fluctuation component (e.g., $\frac{{\rm{\partial }}{u}^{{\rm{{\prime} }}}}{{\rm{\partial }}z}$) measured with the shear probe as

4 $$\varepsilon =\frac{15}{2}\nu \langle {\left(\frac{{\rm{\partial }}{u}^{{\rm{{\prime} }}}}{{\rm{\partial }}z}\right)}^{2}\rangle =\frac{15}{2}\nu {\int }_{0}^{{\rm{\infty }}}{\Psi }_{S}(k)dk,$$

where *v* is kinematic viscosity of water and $\langle {\left(\frac{{\rm{\partial }}{u}^{{\rm{{\prime} }}}}{{\rm{\partial }}z}\right)}^{2}\rangle$ is the variance of the velocity shear fluctuations, the angled brackets indicating averaging over a statistically uniform turbulent patch.

Based on shear probe measurements, the *ε*_*S*_ can be directly obtained by integrating the velocity shear wavenumber spectrum Ψ_*S*_(*k*) over the entire turbulence wavenumber range (i.e. up to the Kolmogorov wavenumber, ${k}_{K}=\frac{1}{2\pi }{\left(\frac{{\varepsilon }_{S}}{{\nu }^{3}}\right)}^{1/4}$). However, because the measured spectrum does not always fully resolve the higher wavenumber range of turbulence, a first estimate of *ε*_*S*_ (denoted by the superscript^*I*^) was obtained by integration within the inertial subrange, i.e., from the lowest resolved wavenumber *k*_*L*_ up to *k*_*U*_ = 0.76*k*_*K*_

5 $${\varepsilon }_{S}^{I}=\frac{15}{2}\nu {\int }_{{k}_{L}}^{{k}_{U}}{\Psi }_{S}(k)dk,$$

where most of the shear variance (i.e., the 95%) resides^41^ (see also Technical Note 28 available at https://rocklandscientific.com). The integration was not extended up to *k*_*K*_ to avoid the spectrum region potentially dominated by instrumental noise, which can bias the integration of the observed spectrum, particularly for *ε*_*S*_ values below ~10 × 10^−8^ m^2^ s^−3^ (ref. ^42^). Similarly, we constrained the upper integration wavenumber *k*_*U*_ with the wavenumber-equivalent of the frequency of anti-aliasing filter (*f*_*AA*_ = 98 Hz). Specifically, the 90% of *f*_*AA*_ was considered to avoid the slight attenuation occurring near *f*_*AA*_, so that *k*_*U*_ = min(0.76*k*_*K*_, 0.9*f*_*AA*_/*W*) cpm. For the typical profiling speed of our dataset, this upper bound is lower than *k* = 150 cpm, which is recommended to safely exclude the region of the measured spectrum affected by excessive spatial response boosting due to Eq. (2). An iterative procedure was followed to account for the dependence of *k*_*K*_ on *ε*_*S*_.

In order to correct for the fraction of the variance that is not resolved by integration, the first estimate ${\varepsilon }_{S}^{I}$ was adjusted upwards using the empirical Nasmyth spectrum^41–43^ as a reference spectrum (hereafter denoted with the superscript *ref*). Specifically, this was done assuming that the variance fraction *α* contained in the measured spectrum in the range of the resolved wavenumbers [*k*_*L*_, *k*_*U*_] is equal to that contained in the empirical Nasmyth spectrum evaluated for ${\varepsilon }_{S}^{I}$ within the same wavenumber range, that is ${\varepsilon }_{S}^{I}=\alpha {\varepsilon }_{S}$ and

6 $${\varepsilon }_{S}^{ref}=\frac{15}{2}\nu {\int }_{{k}_{L}}^{{k}_{U}}{\Psi }_{S}^{ref}(k,\,{\varepsilon }_{S}^{I})dk=\alpha {\varepsilon }_{S}^{I}$$

where ${\Psi }_{S}^{ref}(k,\,{\varepsilon }_{S}^{I})$ is the Nasmyth spectrum evaluated for ${\varepsilon }_{S}^{I}$, and ${\varepsilon }_{S}^{ref}$ is its integral over the range [*k*_*L*_, *k*_*U*_]. The rate of dissipation of TKE, *ε*_*S*_, corrected for unresolved variance was then evaluated as

7 $${\varepsilon }_{S}=\frac{{\varepsilon }_{S}^{{I}^{2}}}{{\varepsilon }_{S}^{ref}}.$$

We refer the reader to the next section for the mathematical definition of the Nasmyth empirical spectrum.

Parallel to the estimate of *ε*, the dissipation rate of thermal variance *χ* was calculated from microstructure temperature observations. Under the assumption of small-scale isotropy, *χ* is determined from

8 $$\chi =6{\kappa }_{T}\langle {\left(\frac{{\rm{\partial }}{T}^{{\rm{{\prime} }}}}{{\rm{\partial }}z}\right)}^{2}\rangle =6{\kappa }_{T}{\int }_{0}^{{\rm{\infty }}}{\Psi }_{T}(k)dk,$$

where *κ*_*T*_ is the molecular thermal diffusivity and $\langle {\left(\frac{{\rm{\partial }}{T}^{{\rm{{\prime} }}}}{{\rm{\partial }}z}\right)}^{2}\rangle$ is the temperature gradient variance, corresponding to the area under a one-dimensional temperature gradient wavenumber spectrum (Ψ_*T*_). Theoretical expressions of the temperature gradient spectrum indicate that it is self-similar and scales with *χ* and the Batchelor wavenumber ${k}_{B}=\frac{1}{2\pi }{\left(\frac{\varepsilon }{\nu {\kappa }_{T}^{2}}\right)}^{1/4}$ (ref. ^44^), so that ${\Psi }_{T}^{ref}={\Psi }_{T}^{ref}(k,\,\chi ,\,{k}_{B})$. Cost function techniques involving fitting of a theoretical spectrum (denoted with the superscript ^*ref*^) to the measured spectrum can therefore be used to calculate the thermal variance dissipation rate (hereafter *χ*_*T*_) and also obtain an alternative (indirect) quantification of the TKE dissipation rate (hereafter *ε*_*T*_), through inference and then inversion of *k*_*B*_. Here we implemented the maximum likelihood estimation (MLE) method proposed by Ruddick *et al*.^45^, using the Kraichnan theoretical spectrum^46^ as fitting spectrum. After preliminary screening of the present dataset and following previous studies^32,47,48^ this theoretical spectrum was found to match observations more closely than the often used Batchelor spectrum^44^. We refer the reader to the next section for the mathematical definition of the Kraichnan theoretical spectrum.

The fitting procedure relied on an iterative optimization procedure with *k*_*B*_ as the only fitting parameter of the theoretical spectrum^45^. *χ* was constrained by observations through the integration of the measured spectrum Ψ_*T*_ over the range [*k*_*L*_, *k*_*U*_], similar to Eq. (5), provided both the viscous-convective and viscous-diffusive subranges (see the next section for details) are resolved. The method required integration after removal of instrument noise, ${\Psi }_{T}^{n}$, to give

9 $${\chi }^{I}=6{\kappa }_{T}{\int }_{{k}_{L}}^{{k}_{U}}\left({\Psi }_{T}\left(k\right)-{\Psi }_{T}^{n}\left(k\right)\right)dk,$$

where ${\Psi }_{T}^{n}$ is the noise spectrum, here calculated following RSI (see Technical Note 40 available at https://rocklandscientific.com), and the ^*I*^ indicates first estimate from integration over a partial wavenumber range. We note that the measured Ψ_*T*_ was corrected for the time response. For consistency, the noise spectrum ${\Psi }_{T}^{n}$ was equally boosted according to Eq. 3. The fraction of unresolved variance outside the integration limits was accounted for by using the same approach delineated in (6)-(7). Because this correction relies on an estimate of the theoretical spectrum, which requires knowledge of *ε*_*T*_, the correction was inserted in the *k*_*B*_ optimization loop, and implemented for each *k*_*B*_ trial. This is an essential modification to the original Ruddick *et al*.^45^ algorithm.

The iterative fitting procedure was performed in three steps, in which *k*_*B*_ is successively refined by narrowing down the search interval^45^. The first iteration served as an exploratory round to identify a preliminary value of *k*_*B*_, which was used to set the lowest integration wavenumber *k*_*L*_ in the second and third iteration steps. Specifically, we followed the procedure out lined by Steinbuck *et al*.^49^ and chose *k*_*L*_ as the maximum of the lowest resolved wavenumber and the upper bound of the inertial-convective subrange *k* = 0.04*Pr*^−0.5^*k*_*B*_, where *Pr* = *ν*/*κ*_*T*_ is the molecular Prandtl number. In addition, possible spectral contamination at low wavenumbers due to internal waves or fine-structure patterns (e.g., stationary density stratified layers) were removed following Luketina and Imberger^50^, i.e., by increasing *k*_*L*_ to the lowest wavenumber where the measured and the theoretical spectra (based on the previous iteration) intersected due to the presence of negative slopes in the measured spectrum. The upper integration limit *k*_*U*_ was defined as the minimum wavenumber among the following four: Batchelor wavenumber *k*_*B*_, the wavenumber where the signal to noise ratio gets smaller than a factor 1.55 (ref. ^39^), the wavenumber corresponding to a 10-fold time response correction (*H*(*f* ) < 0.1 in Eq. 3), the wavenumber equivalent of 90% of the anti-aliasing frequency of the instrument (*f*_*AA*_ = 98 Hz).

The MLE procedure assumes to know the expected statistical distribution of the observations of the temperature spectra. Following Ruddick *et al*.^45^, we assumed that the ratio $d\frac{{\Psi }_{T}}{{\Psi }_{T}^{ref}+{\Psi }_{T}^{n}}$ followed a chi-squared distribution ${\chi }_{d}^{2}$, where *d* is the degrees of freedom of the spectral estimates, depending on spectral technique, window, and averaging methods used. According to ref. ^51^, *d* = 1.9*N*_*f f t*_, where *N*_*f f t*_ is the total (i.e., accounting for overlapping windowing) number of fast Fourier transform segments. In this way, the MLE accounted for the statistical significance of the spectral observations, avoiding any dependency of the resulting estimates on the smoothness of the spectra^52^. The MLE estimate of *k*_*B*_ was obtained by maximizing the log-likelihood function:

10 $$\ell =ln\left({\mathscr{L}}\right)=\mathop{\sum }\limits_{{k}_{i}={k}_{L}}^{{k}_{U}}ln\left[\frac{d}{{\Psi }_{T}^{ref}({k}_{i},\,{k}_{B},\,{\chi }_{T})+{\Psi }_{T}^{n}({k}_{i})}\times {\chi }_{d}^{2}\left(d\frac{{\Psi }_{T}({k}_{i})}{{\Psi }_{T}^{ref}({k}_{i},\,{k}_{B},\,{\chi }_{T})+{\Psi }_{T}^{n}({k}_{i})}\right)\right],$$

where the second term in the right hand side is the chi-square probability density function.

Finally, an alternative estimate of the thermal variance dissipation rate (hereafter *χ*_*ST*_) was calculated using the value of *ε*_*S*_ from the shear probes to directly calculate *k*_*B*_, hence by-passing the iterative fitting procedure described above. Specifically, this value of *k*_*B*_ was used to correct the measured *χ*^*I*^ (Eq. 9) for the unresolved variance above and below the integration limits, similar to (6)-(7). This approach takes full advantage of the fact that the two types of sensors are simultaneously mounted on the instrument (from here the use of the subscript *ST*  ) and is particularly useful in turbulent patches where the profiling speed of the instrument is too high to properly resolve the higher wavenumbers of the temperature gradient spectrum, and thus to accurately determine *k*_*B*_ from spectral fitting.

According to ref. ^45^, *χ*_*T*_ and *χ*_*ST*_ where estimated only when the measured spectrum was significantly above the noise level, i.e. a signal-to-noise ratio larger than 1.3 was required in the integration range [*k*_*L*_, *k*_*U*_]. Some explicative examples of spectral analysis of shear and temperature gradient spectra are reported and commented in the ‘Technical Validation’ section.

### Nasmyth and Kraichnan spectra

The Nasmyth spectrum is an empirical turbulence spectrum that was derived from velocity measurements collected by Nasmyth^43^ in turbulent waters off the west coast of British Columbia. While the Nasmyth spectrum is empirical and not derived from theoretical considerations, it is widely used as a reference of the spectral structure of turbulence in natural waters. The original data were condensed into a single non-dimensional spectrum, which was tabulated by Oakey^41^. More recently, Wolk *et al*.^42^ proposed a fitted form of the dimensionless Nasmyth spectrum that was later updated by RSI (see Technical Note 28 available at https://rocklandscientific.com). Here, we used this most recent form, which reads as

11 $${\widehat{\Psi }}_{S}^{ref}({\widehat{k}}_{S})=\frac{8.05{\widehat{k}}_{S}^{(1/3)}}{1+{\left(20.6{\widehat{k}}_{S}\right)}^{3.715}},$$

where the symbol  ^  indicates a dimensionless quantity and ${\widehat{k}}_{S}=k{\left(\frac{{\nu }^{3}}{\varepsilon }\right)}^{1/4}$ is the dimensionless wavenumber. The corresponding dimensional Nasmyth spectrum is given by

12 $${\Psi }_{S}^{ref}(k)={\left(\frac{{\varepsilon }^{3}}{\nu }\right)}^{1/4}{\widehat{\Psi }}_{S}^{ref}({\widehat{k}}_{S}).$$

**Fig. 2:**
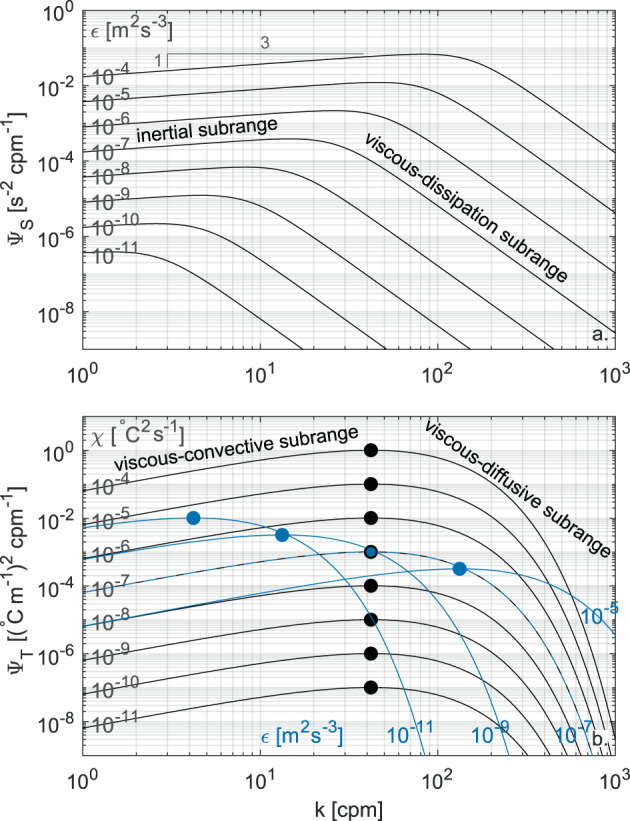
One-dimensional shear (Nasmyth) and temperature gradient (Kraichnan) spectra used as a reference for the processing of the turbulence data. Nasmyth spectra for different values of *ε* (**a**) and Kraichnan spectra for different values *χ* and *ε* fixed at 10^−7^ m^2^ s^−3^ (black lines), and for different values of *ε* with *χ* fixed at 10^−7^ °C^2^ s^−1^ (blue lines) (**b**). The different regions of the spectra are labeled and the maxima of the Kraichnan spectra are depicted with a circle.

The empirical Nasmyth spectrum covers both the range of wavenumbers where velocity fluctuations are not dampened by viscosity (i.e., the inertial subrange predicted by Kolmogorov^53^) and the higher range of wavenumbers where TKE is dissipated as heat by viscosity (i.e., the viscous-dissipation subrange). In the first region the spectrum raises as *k*^1/3^ until reaching a peak, beyond which the spectrum rolls off in the range of wavenumbers affected by viscosity. With increasing *ε*, the Nasmyth spectrum shifts upwards and its peak moves to higher wavenumbers (see Fig. 2a).

The power spectrum of a conserved scalar field (such as temperature) that is passively advected by turbulence in a fluid with a Prandtl number larger than unity (i.e, *Pr* > 1) was first derived by Batchelor^44^. Kraichnan^46^ extended Batchelor’s theory by proposing an alternative (simpler) form of the scalar power spectrum. Under isotropic conditions, the resulting theoretical expression for the Kraichnan dimensionless one-dimensional power spectrum of temperature gradients is

13 $${\widehat{\Psi }}_{T}^{ref}({\widehat{k}}_{T})={\widehat{k}}_{T}\exp (-\sqrt{6q}{\widehat{k}}_{T}),$$

where ${\widehat{k}}_{T}=\frac{k}{{k}_{B}}$ is the dimensionless wavenumber, and *q* is the so-called turbulent parameter. The corresponding dimensional Kraichnan spectrum is given by

14 $${\Psi }_{T}^{ref}(k)=\frac{\chi }{{\kappa }_{T}{k}_{B}}q{\widehat{\Psi }}_{T}^{ref}({\widehat{k}}_{T}).$$

The turbulent parameter *q* is a dimensionless constant, whose value can be estimated experimentally but that is uncertain^48^. Various experimental values of this parameter have been proposed in previous literature, typically varying between 3.4 and 7.9^48,54^. Following ref. ^55^ and according to previous studies^35,54^, here we used *q* = 5.26.

The Kraichnan spectrum rises slowly within the viscous-convective subrange (Ψ_*T*_(*k*) ∝ *k*^1^) until reaching a peak at the wavenumber ${k}_{P}=\frac{{k}_{B}}{\sqrt{6q}}$ (obtained by solving $\frac{d{\Psi }_{T}^{ref}(k)}{dk}=0$). For higher wavenumbers, the spectrum rolls off into the viscous-diffusive subrange. The inertial-convective subrange that develops at wavenumbers smaller than those of the viscous-convective subrange is not covered. Changes in *χ* shift the spectrum vertically, while changes in *ε* shift the spectrum along a −1 slope in log-log space (see Fig. 2b). A reliable estimate of *χ* requires that both the viscous-convective and viscous-diffusive subranges are resolved. Hence, high values of *ε* and profiling speeds may challenge the processing of the data, since under these conditions the viscous-diffusive subrange moves towards right (see Fig. 2b), entering the range of wavenumbers potentially dominated by instrumental noise or affected by large time response correction (see also ref. ^35^).

### Quality metrics

The quality of *ε* and *χ* estimates was checked according to the following metrics. The mean absolute deviation (MAD) between measured and reference spectra was used to identify poorly fitted spectra, both for shear and temperature data. Following^45^, MAD was defined as

15 $$MAD=\frac{1}{N}\mathop{\sum }\limits_{{k}_{i}={k}_{L}}^{{k}_{U}}\left|\frac{\Psi ({k}_{i})}{{\Psi }^{ref}({k}_{i})+{\Psi }^{n}({k}_{i})}-\left\langle \frac{\Psi (k)}{{\Psi }^{ref}(k)+{\Psi }^{n}(k)}\right\rangle \right|$$

where *N* is the number of individual spectral points used for spectral integration and the angled brackets indicate averaging over the range of wavenumbers from *k*_*L*_ to *k*_*U*_. A segment was discarded if MAD was larger than MAD_*c*_ = 2(2/*d*)^0.5^, with *d* the degrees of freedom of the measured spectrum^45^. This threshold corresponds to twice the MAD theoretically expected from a perfect fit, assuming that observations are subject to statistical variability, with the ratio between observed and reference spectra being chi-squared distributed as described above^45^. We note that the noise spectrum Ψ^*n*^ at the denominator was considered only in the case of temperature gradient spectra. In the case of *χ*_*ST*_, an estimate was considered acceptable when contemporaneously the MAD for at least one of the two shear probes and the MAD for the given FP07 probe (i.e., the MAD between measured and theoretical spectra using *k*_*B*_ from the shear probes) were below the rejection threshold. When the MAD for both shear probes was acceptable, *χ*_*ST*_ for both FP07 was calculated using an average of the two *k*_*B*_ value obtained independently from the two shear probes. When only one shear probe passed the quality check, the corresponding *k*_*B*_ value was used for both FP07.

Additional quality criteria were considered only for the estimates of *χ*_*T*_ and *ε*_*T*_ derived from MLE fitting. First, to ensure that the measured spectrum did resolve the change of slope between the viscous-convective and viscous diffusive subrange, the likelihood function of the theoretical spectrum fit (${\mathscr{L}}$) was compared to the likelihood function of a power-law fit (${{\mathscr{L}}}_{pw}$, i.e., a linear fit in a log-log space). Only if the fit to the theoretical curve was significantly better than the power-law fit then the segment was accepted. As a rule, a likelihood ratio *LR* between theoretical and power-law MLE larger than 10^2^ was considered acceptable^45^, *LR* being defined as follows

16 $$LR={\log }_{10}\left(\frac{{\mathscr{L}}}{{{\mathscr{L}}}_{pw}}\right) > 2.$$

Second, to ensure that a sufficient amount of data was used for curve fitting, including the peak of the spectrum and the initial part of the roll off region, only segments with

17 $${k}_{L} < {k}_{P},\quad {\log }_{10}{k}_{U}-{\log }_{10}{k}_{L} > 0.8,\quad {k}_{U} > 2{k}_{P},$$

were accepted^50^. We note that differently from ref. ^50^, in the third condition we used a factor 2 instead of 3 as this was considered enough to safely exclude bad fitted segments. The quality check criterion indicated in Eq. (17) will be hereafter referred to as integration range criterion.

### Other processed variables

Parallel to the processing of the turbulence quantities introduced above, other standard variables were quantified. Salinity (*Sal*) was calculated from the CTD profiles measured with the precise CT sensor, using the empirical equations proposed for Lake Garda^56^

18 $$Sal=27.9+0.72{C}_{20},$$

where *Sal* is expressed in mg l^−1^ and *C*_20_ is water conductivity in *μ* S cm^−1^ at *T* = 20° C. From this, water density, *ρ*, was calculated using the three-order polynomial equation of state proposed by^57^, with water temperature from the precise CT sensor and accounting for the salinity effect (considering the haline contraction coefficient of calcium bicarbonate *β* = 0.807 × 10^−3^ kg g^−1^, bicarbonate and calcium ions contributing more than 80% of Lake Garda salinity^56^).

Following Thorpe and Deacon^58^, the length scale (*L*_*T*_) representative of the vertical size of turbulent overturns in a stratified water column was determined by implementing a standard sorting procedure of the water density profile. Specifically, the observed density profiles were reordered into the corresponding stable profiles and the length *L*_*T*_ was determined as the root mean square of the vertical displacements (*d*_*T*_) resulting from the resorting procedure

19 $${L}_{T}={\left\langle {d}_{T}^{2}\right\rangle }^{1/2},$$

where the angled brackets indicates averaging, and *L*_*T*_ and *d*_*T*_ have the units of m and are typically referred to as Thorpe length and Thorpe displacements, respectively. The Thorpe scale was evaluated locally for each segment where the turbulence quantities were calculated.

For each segment, the local background stability of the water column was quantified with the Brunt-Väisälä frequency

20 $${N}^{2}=-\frac{g}{{\rho }_{0}}\left\langle \frac{\partial \rho }{\partial z}\right\rangle ,$$

where *g* is the gravitational acceleration (9.81 m s^−1^), *ρ*_0_ is a reference freshwater density (1000 kg m^−3^), and $\left\langle \frac{\partial \rho }{\partial z}\right\rangle$ is the average vertical gradient of the local water density profile obtained by linear fitting the density data in that segment. When the local Thorpe scale was larger than the predefined segment thickness (3 m), the vertical density gradient was evaluated over a longer segment with thickness equal to *L*_*T*_. In order to calculate a value of *N*^2^ representative of background stratification, the sorted stable water density profile was used^59,60^.

Because of the technical limitations to measure turbulent fluxes directly, in lake and ocean studies those are often parameterized with a turbulent vertical diffusivity (*K*). When *ε* estimates are available, *K* can be quantified using the Osborn relation^7^

21 $${K}_{Osborn}=\Gamma \frac{\varepsilon }{{N}^{2}},$$

The parameter Γ, or mixing efficiency, describes the fraction of the dissipated energy that is used to work against the stable stratification and lift heavy fluid, i.e. to do mixing. While Γ ≈ 0.2 is typically used as a standard value of the mixing efficiency, based on the present dataset Γ = 0.28 has been quantified as a more appropriate value for Lake Garda (see the details and discussion in the ‘Usage Notes’ section).

Measurements of the thermal variance dissipation rate, *χ*, which are typically scarcer than *ε* measurements, allow for an alternative, more direct estimate of the diapycnal diffusivity according to the Osborn and Cox model^8^,

22 $${K}_{Osborn-Cox}=0.5\chi {\left\langle \frac{\partial T}{\partial z}\right\rangle }^{-2},$$

where *χ* is obtained from microstructure measurements and $\left\langle \frac{\partial T}{\partial z}\right\rangle$ is the average temperature gradient obtained by linear fitting the temperature data within a segment. Temperature from the FP07 sensors was used due to their higher resolution, which allowed for more accurate estimates of the temperature gradients, especially under weakly stratified conditions. Similar to the definition of *N*^2^, the sorted temperature profile was used as representative of background stratification and the temperature gradients were evaluated over a longer segment of thickness *L*_*T*_ when this was larger than the predefined segment thickness.

The diapycnal diffusivity defined as in the two models described above was computed locally for each segment, and for all the available estimates of *ε* and *χ*. This resulted in a maximum value of eight estimates per segment provided that two shear probes and two fast-response thermistors were used and that all the *ε* and *χ* fits met the quality checks.

## Data Records

The dataset is archived as a collection of raw files and processed files. Each file contains the set of vertical profiles measured in a monitoring site in a single day, and is identified by an increasing ID number. A.csv table summarizing the list of records with the associated ID number, measurement date and time, weather conditions, Secchi depth, and general notes is provided in the dataset for reference.

The raw files (.P) provided by the instrument are complemented by the instrument configuration file (.cfg), which contains comprehensive instrument’s metadata and probes’ calibration parameters needed for independent processing and of the measured records and reproducibility of the analysis.

The processed data are provided both as NetCDF files (a common, self-describing, portable format for scientific data) and as.mat files (a proprietary Matlab data format that can also be opened using open-source platforms such as Python, R, and GNU Octave). Each processed file contains the turbulence, water quality, and physical quantities listed in Online-only Table 1. The archived variables are divided into three groups according to their resolution: FAST for data at 512 Hz, SLOW for data at 64 Hz, and BINNED for quantities computed over 3 m segments. For a given vertical microstructure profile, each group of variables is saved in a separate NetCDF file (for a total of 606 profiles × 3 groups = 1818 NetCDF files). As for.mat files, each group of variables is stored as an independent structure in the same vertical profile file (for a total of 606.mat files). The meteorological records (METEO) merge the data from the on-board thermo-hygrometer and meteorological station, and are also provided as NetCDF and.mat files. The dataset is completed by a README file describing the content of the dataset, a table listing and describing the available observables, and the key scripts (in Matlab) for the analysis of the turbulence spectra.

The data are accessible via Dryad^61^ at the following link: 10.5061/dryad.nk98sf7sk.

## Technical Validation

### Quality screening and inter-sensor cross-validation

The technical validation of the dataset followed a two-step approach. In the first step, we discarded for each sensor all segments for which the turbulence estimates did not meet the quality metrics introduced above. Overall, a total of ~ 39000 estimates of *ε* and *χ* were obtained for each sensor. Around 90 % of *ε*_*S*_ estimates, 77 % of *ε*_*T*_ and *χ*_*T*_ estimates, and 75 % of *χ*_*ST*_ estimates passed the quality check based on the quality metrics (see Table 1).

**Table 1:** Number of total and quality check passed estimates of TKE and temperature variance dissipation rates.

Quantity	N estimates	N good estimates	% good estimates
*ε*_*s*1_	39148	36233	92.6%
*ε*_*s*2_	39162	34835	89.0%
*χ*_*T*1_, *ε*_*T*1_	39330	30217	76.8%
*χ*_*T*2_, *ε*_*T*2_	39330	30906	78.6%
*χ*_*ST*1_	39167	29370	75.0%
*χ*_*ST*2_	39167	30080	76.8%

**Fig. 3:**
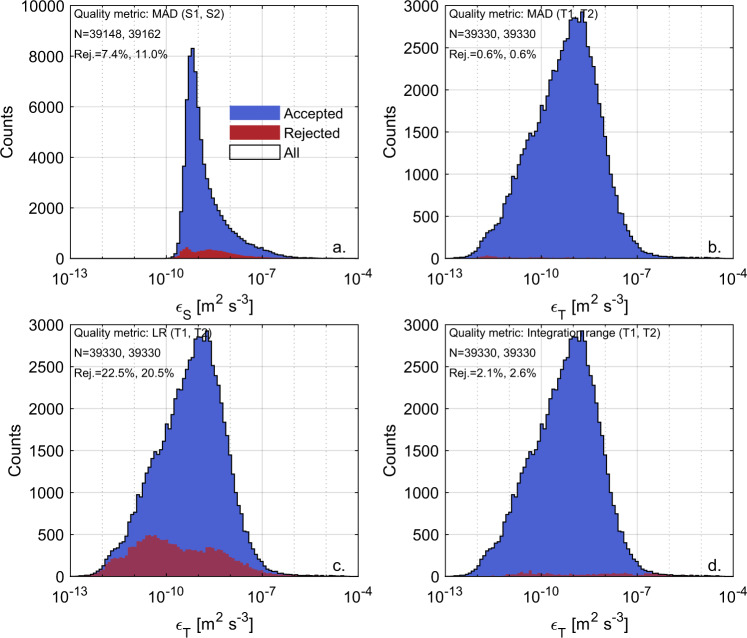
Density distribution of accepted and rejected *ε* estimates according to the metrics used for quality check. Histograms of accepted (blue) and rejected (red) *ε*_S_ according to the mean absolute deviation (MAD) metric (**a**), and of accepted and rejected *ε*_*T*_ according to the MAD metric (**b**), the likelihood ratio (LR) metric (**c**), and the integration range criterion (**d**). In each plot, the overall distribution of *ε* estimates is shown with a black contour line. The histograms merge all estimates from shear probes S1 and S2 (**a**) and FP07 T1 and T2 (**b**–**d**), while the number of total estimates (N) and the percentage of accepted estimates for each sensor are reported at the top left corner of each subplot.

The probability density distributions of *ε* estimates are shown in Fig. 3 for shear probes (panel a) and FP07 sensors (panels b-d). Accepted and rejected values according to each quality metric are shown separately. As for the case of *ε*_*S*_ (panel a), the few (i.e., ~10%) rejected values are mainly found for *ε*_*S*_ < 10^−8^ m^2^ s^−3^, corresponding to the range where the observed spectrum is expected to be mostly affected by instrumental noise^42^.

In the case of *ε*_*T*_, nearly all rejected estimates is attributable to the LR metric (i.e., ~20% of all estimates, panel c). Although the integration range criterion described in Eq. (17) alone only rejects ~2.5%, it turned out to be decisive for rejecting the poor estimates in the range *ε* > 10^−7^ m^2^ s^−3^ that are not excluded by the other quality metrics (panel d). Conversely, the quality check of *ε*_*T*_ is insensitive to the MAD criterion, which alone excludes less than ~1% of values and does not reject additional values with respect to the other criteria (panel b). Similarly to ref. ^45^, we therefore found that the likelihood ratio LR is a highly informative indicator of poor spectral fits, but differently, the same was not found to be true for MAD. Our results further indicated that including a criterion on the integration range (see Eq. (17)) is more effective than the LR metric to safely reject segments where the observed spectrum did not resolve the sharp roll off of the theoretical spectrum, in agreement with previous reports^50^.

**Fig. 4:**
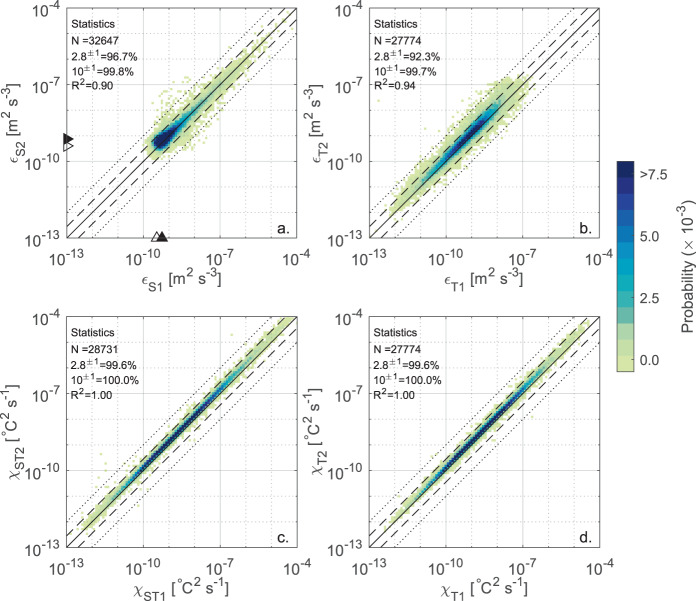
Cross-validation between sensors of the same type. Heatmap plot showing the probability density distribution of the estimates of *ε*_*S*_ from shear probes 1 and 2 (**a**), *ε*_*T*_ from FP07 1 and 2 (**b**), *χ*_*ST*_ from FP07 1 and 2 (**c**), and *χ*_*T*_ from FP07 1 and 2 (**d**). The probability density distribution (heatmap) is calculated by dividing the range of variability of the dissipation rates into 100 logarithmically spaced intervals. Only values passing the quality checks were analyzed. The number of analyzed data (N) and the percentage of estimates falling within a factor of 2.8 (dashed line) and 10 (dotted line) of each other are reported at the top left corner together with the coefficient of determination *R*^2^. The 1:1 line (solid line) is also shown, along with the mode (filled triangle) and 5th percentile (empty triangle) for shear probes estimates used to quantify the noise floor.

In the second step of the data validation procedure, we cross-validated the *ε* and *χ* estimates across the four sensors, considering only the segments not rejected by the quality metrics screening. In Fig. 4 the estimates of *ε* and *χ* are compared between sensors of the same type: i.e., shear probe 1 (S1) vs. shear probe 2 (S2), and FP07 1 (T1) vs. FP07 2 (T2). The cross-validation is graphically presented as a heatmap plot showing the probability density distribution of the estimates. The percentage of estimates that are scattered within a factor of 2.8 and 10 are reported in the corresponding panels, along with the coefficient of determination *R*^2^ of a linear regression model. The factor 2.8 follows from error propagation theory, according to the fact that, based on experimental evidence, a factor of 2 is considered as the natural variability of turbulence measurements^39,41,62,63^. Hence estimates from independent probes could be scattered within a factor of $\sqrt{\left({2}^{2}+{2}^{2}\right)}=2.8$. The factor of 10 is considered as the highest reasonable threshold for coherent measurements from independent sensors.

Figure 4 indicates high coherence between turbulent quantities estimated independently from probes of the same type. In all cases, more than 90% of estimates lie within the expected statistical variability, and > 99% within a factor of 10. The consistency between independent measurements is particularly high for *χ* (more than 99% of estimates within a factor of 2.8, panels c and d), while it is slightly lower for *ε*, particularly for the FP07 sensors (panel b), compared to the shear probes (panel a). We attribute the larger scatter found for *ε*_*T*_ to i) its indirect estimation through MLE fitting procedure, compared to the direct spectral integration used to calculate *ε*_*S*_ and *χ*_*ST*_, and ii) to the potentially incomplete resolution of the viscous-diffusive subrange of the measured temperature gradient spectra due to the time response limitation.

**Fig. 5:**
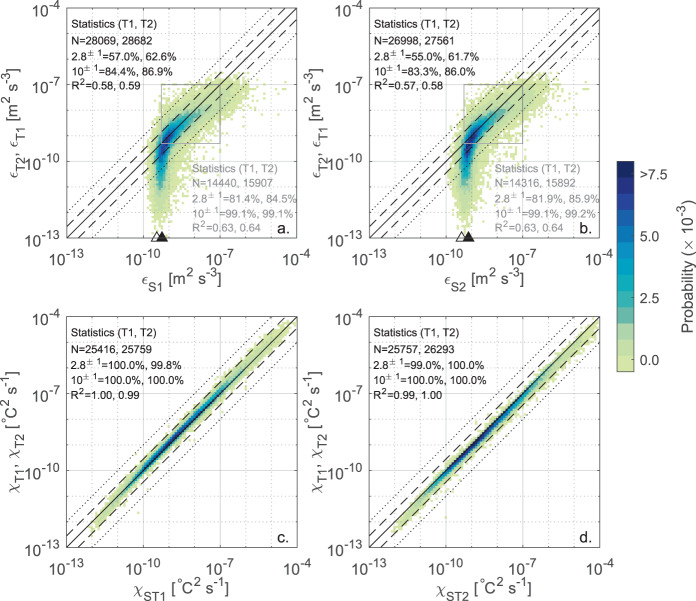
Cross-validation between shear probes and FP07. Heatmap plot showing the probability density distribution of the estimates of *ε* from shear probe 1 (**a**) and shear probe 2 (**b**) versus FP07 1 and 2, and of the estimates of *χ* from combined shear probes and FP07 1 (**c**) and FP07 2 (**d**) versus FP07 1 and 2. The probability density distribution (heatmap) is calculated by dividing the range of variability of the dissipation rates into 100 logarithmically spaced intervals. Only values passing the quality checks where analyzed. The number of analyzed data N and the percentage of estimates falling within a factor of 2.8 (dashed line) and 10 (dotted line) of each other are reported at the top left corner together with the coefficient of determination *R*^2^, for each shear-FP07 pair separately. The text in gray refers to the statistics of the *ε* estimates falling within the range 5 × 10^−10^−10^−7^ m^2^ s^−3^ (gray rectangle). The 1:1 line (solid line) is also shown, along with the mode (filled triangle) and 5th percentile (empty triangle) for shear probes estimates used to quantify the noise floor.

The same comparison described above is shown across different types of sensors in Fig. 5: i.e., S1 vs T1 and T2, and S2 vs T1 and T2. The figure confirms the high coherence of *χ* estimates (panels c and d), with all the points contained within a factor of 10. Also, more than 99 % of occurrences lay within a factor of 2.8 when comparing shear probes and FP07. The noticeable matching is partially motivated by the fact that *χ*_*ST*_ estimates result from combining information from both shear probes and fast-response thermistors. Differently, the distributions of *ε* show a clear asymmetry between shear probes and FP07 estimates (panels a and b), due to a visible lower limit detection of shear probes that concentrates estimates within a narrow range from 10^−10^ m^2^ s^−3^ to 10^−9^ m^2^ s^−3^. As a result, only less than 60% of estimates are bounded within the factor of 2.8 of each other and about 85% within a factor of 10. The reasons for this discrepancy between *ε* estimates is discussed in the next section.

### Technical constraints on the use of shear and temperature sensors

The estimates of *ε* from shear probes and FP07 suffer from some technical limitations inherent in the respective sensors. This clearly emerges when looking at the asymmetries between the density distributions in Fig. 3 and the heatmap plots in Fig. 4a,b, and even more explicitly when looking at the one-to-one plots in Fig. 5a,b.

#### Shear probes’ noise floor

**Fig. 6:**
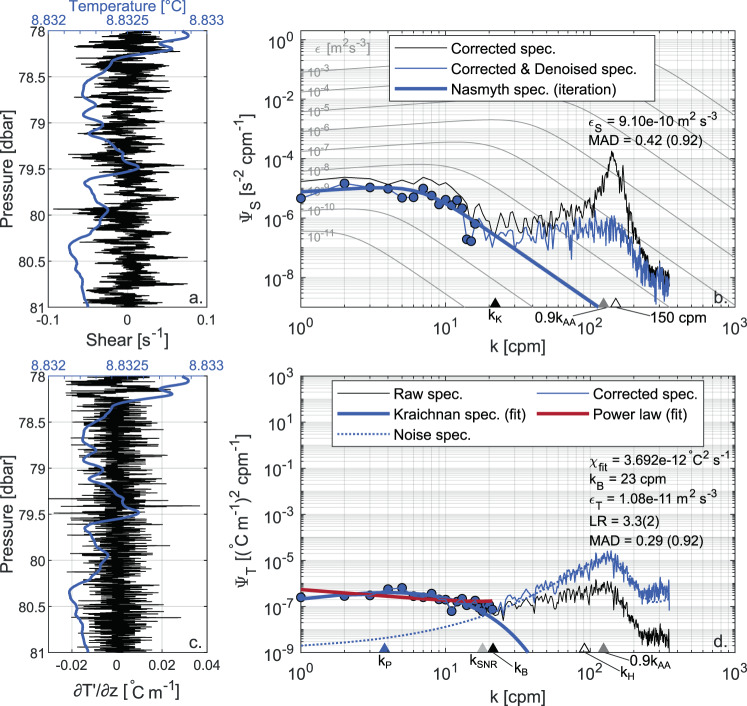
Comparison between shear and temperature gradient spectra, highlighting the noise floor affecting shear probe measurements. The shear signal and the corresponding spectral analysis are shown in panels a and b, while the temperature gradient (∂*T*′/∂*z*) signal and the corresponding spectral analysis are shown in panels c and d. Panels a and c also show the temperature profile (blue line) in the segment (fast-response thermistor T1, smoothed with a moving average with a 100-scan window). The estimated turbulence quantities and the quality metrics are reported in the figure along with the relevant wavenumbers: Kolmogorov (*k*_*K*_), Batchelor (*k*_*B*_), peak of the Kraichnan spectrum (*k*_*P*_), 90% of the anti-aliasing filter (0.9*K*_*AA*_), wavenumber for which the signal to noise ratio gets smaller than 1.55 (*k*_*SNR*_), and for which the temperature gradient spectrum is corrected by more than a factor of 10 (i.e., where *H*(*f* ) < 0.1, *k*_*H*_). Panel b shows the observed shear spectra after spatial response correction (thin black line) and removal of coherent noise (thin blue line), compared with the Nasmyth empirical spectrum resulting from the iterative integration procedure (thick blue line). Panel d shows the observed temperature gradient spectra obtained from raw data (thin black line) and after time response correction (thin blue line), the fitted Kraichnan theoretical spectrum (thick blue line), the fitted power-law (thick red line), and the sensor noise spectrum (dotted blue line, after time response correction for consistency). In both panels, the range of integration of the observed spectra is indicated with filled circles. The comparison between the shear and temperature gradient spectra highlights that in the first case *ε* is overestimated by more than two orders of magnitude. This overestimation is attributed to the noise floor of the shear probes. The data refer to the segment from 78 to 81 m depth of the first profile in file DAT_042 acquired on 10 March, 2017 (data from S2 and T2 respectively).

The main reason for this discrepancy is that *ε*_*S*_ estimates are lower bounded by the sensitivity limit of the shear probes, which results in masking the environmental shear signal by electronic measurement noise in low energetic environments^64^. This noise floor biases the integration of the observed spectrum towards overestimated values, and artificially skews the *ε*_*S*_ distribution towards larger values than those actually occurring in the fluid. Figure 6a,b shows the spectral analysis of an observed shear spectrum. The biasing effect of the noise floor emerges only when it is compared to the spectral analysis of a temperature gradient spectrum measured in the same segment (Fig. 6c,d). Indeed, the overall shape of the observed shear spectrum is good, and there is not explicit evidence motivating the rejection of the segment. This is an eloquent example of the convenience of combining shear and fast-response thermistors for quality control purposes. Scheifele *et al*.^64^ suggested to calculate the mode of the *ε*_*S*_ estimates as a way to quantify the noise floor. When applying this approach to our dataset, we obtained values of 5 × 10^−10^ m^2^ s^−3^ and 8 × 10^−10^ m^2^ s^−3^ for S1 and S2, respectively (filled triangles in Figs. 4 and 5). Since these values are close to the range of values containing the bulk of *ε*_*T*_ estimates from FP07 sensors (see Fig. 3b–d), we wondered that for the dataset at hand the mode could bias the quantification of the shear probes’ noise floor towards the most recurrent values of *ε* for the investigated system. We therefore calculated also the 5th percentile of the *ε*_*S*_ estimates as a safer indicator of the lowest detection limit of shear probes, obtaining slightly lower values of 3 × 10^−10^ m^2^ s^−3^ and 4 × 10^−10^ m^2^ s^−3^, for probes S1 and S2, respectively (open triangle in Figs. 4 and 5). Overall, these values are consistent with previous studies based on similar loosely tethered profilers^42,65,66^ which reported a noise floor of *O*(10^−10^) m^2^ s^−3^. We note that the rejection of a significant portion of *χ*_*St*_ estimates can be related to the noise floor effect. When the “true” value of *ε* is below the shear sensor detection limit, the higher *ε*_*S*_ estimate used to fit the observed temperature gradient spectrum is likely to result in an unacceptable MAD. This is confirmed by the statistics of *χ*_*St*_ rejections, whereby the median of rejections is clustered at *ε*_*S*_ ~ 8 × 10^−10^ m^2^ s^−3^.

#### FP07 time response limitation

**Fig. 7:**
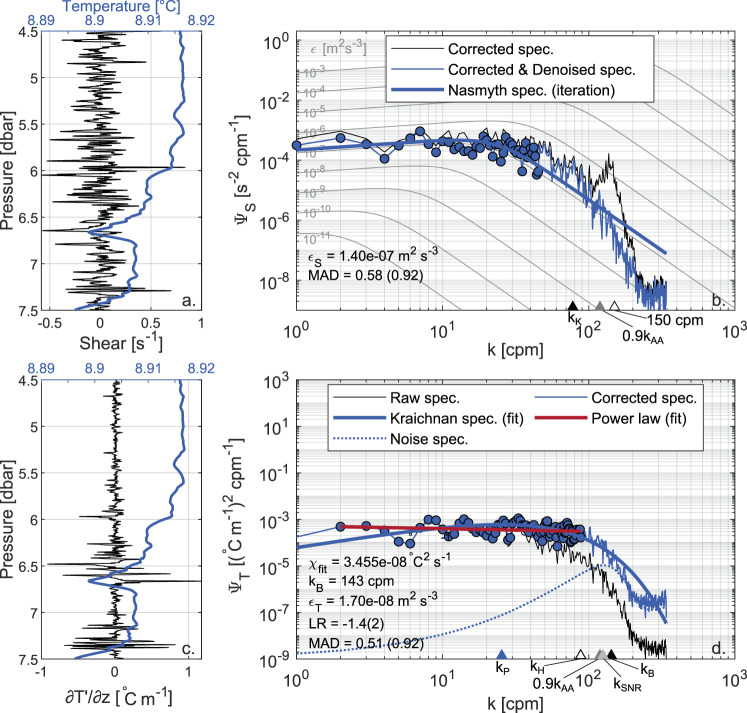
Comparison between shear and temperature gradient spectra, highlighting the time response limitations affecting FP07 measurements. For the details of the figure see the caption of Fig. 6. After time response correction, the shape of the observed temperature gradient spectrum is flattened and the estimated value of *ε* is smaller than that resulting from shear probes. As an effect of the time response correction, the roll off of the spectrum is not properly resolved and the LR metric rejects the spectral fitting based on the Kraichnan theoretical spectrum as it is outperformed by a power-law fitting. The data refer to the segment from 4.5 to 7.5 m depth of the fourth profile in file DAT_181 acquired on 23 March, 2018 (data from S1 and T2 respectively).

The one-to-one comparison between *ε* estimated from shear probes and FP07 (Fig. 5a,b) also shows (slighter) departure from the 1:1 line at the upper tail of the distribution. In addition to that, a clear cut-off is visible for *ε*_*T*_ = *O*(10^−7^) m^2^ s^−3^, with few acceptable *ε*_*T*_ estimates above this threshold according to the quality metrics. The reason is found in the time response correction applied to the FP07 thermistors (see Eq. (3)), whereby the observed temperature gradient spectrum is largely corrected at the highest wavenumbers for a profiling speed of ~0.75 m s^−1^. Hence, for high-energy turbulent patches, the roll off of the spectrum is not properly resolved and i) the estimate of *ε*_*T*_ is not accurate (i.e., it is typically underestimated) and/or ii) its rejection is more likely. This suggests an upper limit for acceptable spectral fits at *ε*_*T*_ = *O*(10^−7^) m^2^ s^−3^. An example of this effect is shown in Fig. 7, where the spectral analysis of an observed temperature gradient spectrum is compared to that of a shear spectrum measured in the same segment. In this specific case, the time response correction rises the high wavenumber part of the temperature gradient spectrum, flattening it down. Despite this correction, *ε*_*T*_ is underestimated by one order of magnitude compared to *ε*_*S*_. In addition to that, the Kraichnan theoretical spectrum fitting is outperformed by a power-law fitting, hence resulting in a rejected segment.

We are aware that using a lower profiling speed would have allowed better resolving the viscous-diffusive subrange of temperature gradient spectra. Indeed, analogous loosely tethered profilers mounting fast-response thermistors are typically operated at profiling speeds between 0.1 m s^−1^ and 0.4 m s^−1^ (refs. ^12,21,23,67,68^). However, we found that the a profiling speed of ~0.75 m s^−1^ was a reasonable compromise between optimizing shear and fast-response thermistors performance: the ability of shear probes to be used in medium- to high-energy environments (i.e., *ε* > 5 × 10^−10^ m^2^ s^−3^) compensated the time response limitations of the FP07 sensors. In turn, the FP07 sensors adequately resolved medium- to low-energy patches (i.e., *ε* < 10^−7^ m^2^ s^−3^) where shear measurements were precluded by the noise floor of the probes. In the overlapping region between *ε* = 5 × 10^−10^ m^2^ s^−3^ and *ε* = 10^−7^ m^2^ s^−3^, both types of sensors could be safely used and provided good cross validation scores, with more than 80% of estimates scattered within a factor of 2.8 and 99% within a factor of 10 (see gray box and text in Fig. 5). Overall, this indicates high coherence between the independent estimates obtained from shear probes and FP07 sensors, and suggests that the adopted profiling speed was appropriate to span the wide range of turbulence intensities observed in the lake, once the turbulence estimates were properly interpreted in light of the limitations of the fast-response probes.

**Fig. 8:**
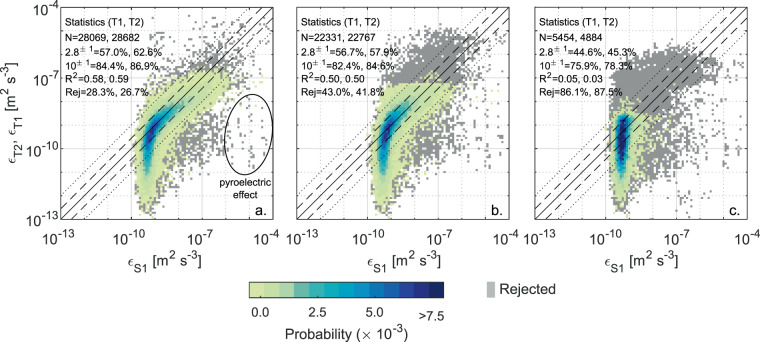
Cross-validation between shear probes and FP07 for three different time response corrections. Heatmap plot showing the probability density distribution of the estimates of *ε* from shear probe 1 versus FP07 1 and 2, according to the time response correction described in the Methods section (**a**), and the approaches proposed by^36^ (**b**) and by^40^. The estimates rejected at least for one of the sensors are depicted in gray. For the details of the figure see the caption of Fig. 5. As a side comment, we note that part of the rejected estimates at the right of the 1:1 line are ascribable to the pyroelectric effect, whereby *ε*_*S*_ is skewed to higher values compared to *ε*_*T*_ (see text and circle in subplot (**a**)).

When using high profiling speeds, the time response correction applied to the temperature-gradient spectra are critical for the calculation of *ε*_*T*_. There is a large spread of corrections proposed in the literature depending on the shape of the transfer function (double-pole vs single pole), on the nominal sensor time response (*τ*_0_), and on the dependency of the effective time response (*τ*) on the profiling speed. In order to seek the best suitable correction for our set-up, we evaluated a set of different possibilities by using *ε*_*S*_ values above the noise as a reference. A synthesis of this evaluation is shown in Fig. 8 for three different time response correction approaches: the one used in the present analysis (Fig. 8a), and those proposed by Vachon and Lueck^36^ (Fig. 8b) and by Sommer *et al*.^40^ (Fig. 8c). According to ref. ^36^, the effective time response *τ* depends on the same velocity scaling used in the present study, but with different parameters, *τ*_0_ = 4.1 × 10^−3^ s and *γ* = −0.5. In addition, a double pole transfer function is used to correct the observed temperature gradient spectrum, which is similar to Eq. (3) but with the right hand side raised to the power of 2. Similarly, Sommer *et al*.^40^ proposed the use of a double pole transfer function, with a fixed *τ* value of *τ* = 10 × 10^−3^ s, independent of the mean flow velocity. Due mainly to the use of the double pole function, both methods resulted in a stronger correction than that proposed here.

The different performance of the three approaches is glaring: while the milder correction used here yielded less than ~30% of rejected estimates (considering the combined rejection from S1 and either T1 or T2), the other methods yielded higher rejection rates of more than ~40% and ~85%, respectively. With the intermediate correction^36^, a clear cut-off is visible for *ε*_*T*_ = *O*(3 × 10^−8^) m^2^ s^−3^, and a general overestimation of *ε*_*T*_ is appreciable in the range between 10^−9^ and 10^−8^ m^2^ s^−3^. When the more severe correction^40^ is used, the cut-off decreases to *ε*_*T*_ = *O*(2 × 10^−9^) m^2^ s^−3^, close to the noise floor of shear probes, thus preventing from evaluating any reasonable correlation between the two different types of sensors. We highlight that Sommer *et al*.^40^. derived their correction from observed differences in the statistical distributions of interface thicknesses of the double-diffusive staircases in Lake Kivu, aimed at reconstructing the “true” temperature signal. However, when used for traditional spectral fitting in the viscous-convective and viscous-diffusive subranges of the temperature gradient spectra, our results clearly show that this correction provides undesirable results. Previous applications of this strong correction were reported for example in ref. ^35^, but for spectral fitting to the inertial-convective subrange, which develops at wavenumbers where the correction has little impact up to *ε*_*T*_ = *O*(10^−5^) m^2^ s^−3^ (see Fig. 1 in ref. ^35^). The same correction was used also to process microstructure measurements from a glider^64^, but in a low energetic environment, thus restricting strong corrections of the measured signal, and in presence of a lower shear probe noise floor (as is typical in gliders compared to loosely tethered profilers^42,63–65^) that allowed for proper comparison between *ε*_*S*_ and *ε*_*T*_ estimates. We acknowledge that the results summarized in Fig. 8 may be partially affected by the relatively high profiling speed used here, thus care should be taken when comparing these data with other turbulence datasets in lakes (which often use lower profiling speeds). On the other hand, based on the above considerations we also caution users against the possible effects of using aggressive correction when operating loosely tethered microstructure profilers in moderate-to-high energetic environments, and suggest that a careful inter-comparison analysis including milder approaches should be undertaken.

#### Pyroelectric effect

**Fig. 9:**
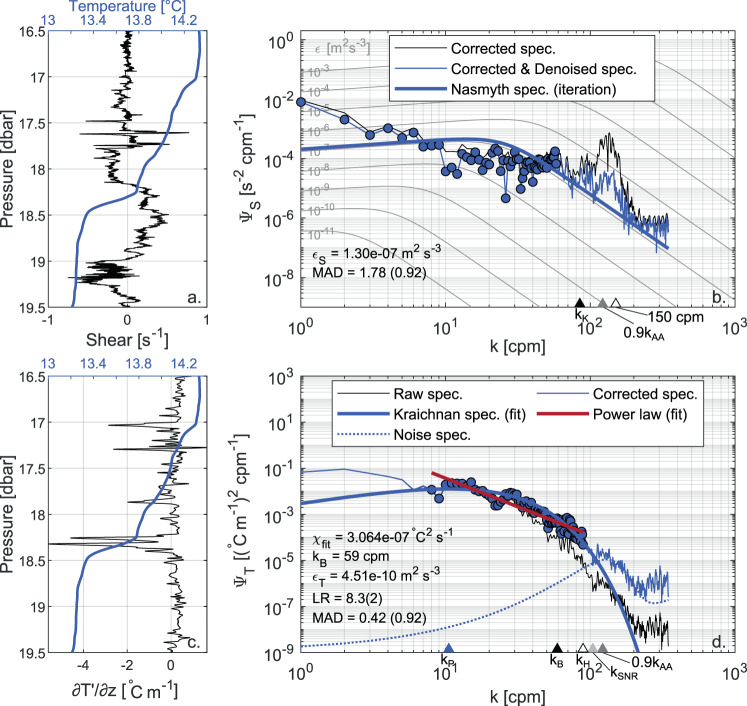
Comparison between shear and temperature gradient spectra, highlighting the pyroelectric effect affecting shear probe measurements. For the details of the figure see the caption of Fig. 6. In correspondence of a sharp temperature gradient, the shear signal presents a sine-like perturbation (**a**) due to pyroelectric effect. This disturbance prevents from measuring a good shear spectrum (**b**), thus resulting in a rejected segment. The signature of the same sharp temperature gradient is also visible in the temperature gradient spectrum, which however is removed following the approach proposed by^50^. The data refer to the segment from 16.5 to 19.5 m depth of the first profile in file DAT_109 acquired on 13 July, 2017 (data from S2 and T2 respectively).

An anomalous shear signal was occasionally recorded when sampling across a sharp thermocline. The sine-like spike presented a much lower frequency compared to the ambient shear signal (Fig. 9a) and was attributed to the pyroelectric effect, i.e. the generation of an electric charge on the piezoceramic shear probe due to the sudden change of temperature at the thermocline. These anomalous signals prevented the *ε*_*S*_ spectral calculation since the observed spectrum did not correspond to the expected Nasmyth spectrum (Fig. 9b). Essentially all occurrences of such pyroelectric disturbance were automatically rejected by the quality check control based on the MAD quality metric. Since the pyroelectric effect is specific of the shear probes, the estimate of *ε*_*T*_ based on the FP07 sensors was not affected (Fig. 9c,d). However, the temperature gradient spectra measured within sharp thermoclines were often contaminated at low wavenumbers by the fine-structure pattern associated with density stratification (Fig. 9d). This contamination was effectively identified and removed following the procedure outlined in ref. ^50^ and described in the previous sections. Based on the acquired dataset, we determined that the median of the maximum local temperature gradient among segments affected by the pyroelectric effect was larger than 0.8° C m^−1^, evaluated with the precise CT sensor. Because comparably sharp thermoclines are common in lakes in summer (the authors observed similar effects also in lakes Geneva and Zurich), attention is drawn to the fact that the pyroelectric effect may result in a general limitation for *ε* measurements in these stratified environments. All the profiles affected by pyroelectric effect were measured in June and July and the anomaly was observed between 18 and 23 m depth (values corresponding to the 25th and 75th percentiles of the affected depths).

## Usage Notes

The detailed analysis of the data is expected to provide key information to characterize the turbulence structures in lakes, where the problem of tuning parameters in turbulence closures for numerical models using microstructure profiling did not advance significantly after some seminal contributions^69–71^. With specific reference to Lake Garda, this dataset will support scientific research on turbulent mixing and allow to improve the performances of a recently developed three-dimensional thermo-hydrodynamic model^30,72,73^, with possible applications to various transport processes and, in particular, to a more accurate estimation of the vertical diffusive fluxes affecting deep mixing.

The dataset is characterized by an unusual spatial and temporal extension. Four different sites were sampled regularly every month, all located in the northern narrow part, but in different locations: pelagic, demersal, and wind-sheltered bay zones. The systematic sampling along a lateral transect is a valuable component of the database, which can be used to get in-depth insights on the lateral heterogeneity of turbulence. This aspect is particularly interesting for this elongated lake, where the presence of periodic, strong longitudinal winds has been shown to generate significant lateral flows due to the Coriolis acceleration^30^. This dataset is therefore ideal to investigate if and to what extent the resulting coastal up- and down-wellings determine different turbulence patterns between the two coasts, and between coastal and pelagic zones. In this respect, the availability of spatially distributed meteorological data acquired at the fieldwork boat is particularly relevant for the analysis. This kind of information is scarce on lakes and is useful for a proper interpretation of the turbulence profiles. Furthermore, once integrated with the data provided by the existing ground stations, it offers the attractive possibility to investigate the spatial heterogeneity of weather conditions over the lake. In addition to that, the acquisition of data during different weather conditions such as the typical alternating lake-valley breezes, strong northerly Föhn wind events, winter frosts due to cold atmospheric fronts, allows to investigate turbulence structures driven by different external factors, complementing the existing literature on the topic^5,74–76^ with exceptionally available year-round, multi-site data. Finally, the dataset is completed with a 24 h fieldwork campaign executed in May 2018, during which the reference transect was monitored in continuous, day and night. These data can be used for a first characterization of the subdaily and day-night mixing variability.

From a methodological perspective and thanks to the combined use of airfoil shear probes and fast-response thermistors, this dataset can contribute to better understanding the performance of these sensors when measuring turbulence quantities varying over several orders of magnitude. The analysis already provided evidence about the possibility to challenge the use of the fast-response thermistors with high profiling speed (i.e., ~0.75 m s^−1^), still obtaining good estimates of *ε*_*T*_. Under these profiling conditions, we suggest that the ideal range of application of the two type of sensors is *ε* > 5 × 10^−10^ m^2^ s^−3^ for shear probes (lower limited by the noise floor of the sensors) and *ε* < 10^−7^ m^2^ s^−3^ for FP07 (upper limited by sensor’s time response). In the overlapping region between *ε* = 5 × 10^−10^ m^2^ s^−3^ and *ε* = 10^−7^ m^2^ s^−3^, both types of sensors could be safely used, thus providing a wide range of turbulent mixing conditions for inter-sensor cross-validation. Future microstructure measurements can take full advantage of the experience synthesized in these data to further optimize the trade-off between an operationally efficient profiling speed and reliable *ε* estimates.

Specifically, the dataset can be used to contribute to the long-lasting debate around the best frequency response correction to be applied to fast thermistor sensors in highly energetic environments^35,37,39,40^, offering a rich amount of data over which implementing systematic time response correction analyses. The key results of the exploratory sensitivity analysis performed here are summarized in Fig. 8 and discussed in the previous section, aimed at providing a practical reference to the community of turbulence microstructure profiler users and stimulus for further research.

Also, the present dataset could be used to advance in general open questions in observational turbulence estimates in natural waters. In the lack of microstructure measurements, TKE dissipation rates are often derived from a turbulence length-scale (*L*_*O*_) and a measure of stratification (*N*, see Eq. 20), as follows

23 $$\varepsilon ={L}_{O}^{2}\,{N}^{3}.$$

This equation is the definition of the Ozmidov length-scale (*L*_*O*_), which represents the upper limit eddy size in the inertial turbulent subrange. However, *L*_*O*_ is not measured directly and it is often replaced by some fraction of the Thorpe scale, i.e. *L*_*O*_ ≈ 0.8*L*_*T*_ (ref. ^77^). As already mentioned before, the Thorpe scale (*L*_*T*_ is an empirical measure of turbulent vertical overturns that can be obtained from a careful operation of regular CTD profilers or moored temperature measurements. For this reason, the length-scale method is popular across a wide range of ocean and lake studies^10,11,78,79^. However, it is increasingly recognized that a simple scaling between *L*_*T*_ and *L*_*O*_ may not be generally applicable, as the ratio between the two scales varies across the different stages of a turbulent event^80^.

Since the seminal work by Osborn^7^, a value of the mixing efficiency Γ ≈ 0.2 has been used as the default value to calculate diapycnal diffusivity (see Eq. (21)) in a large number of studies. Indeed, there is empirical evidence that Γ from field measurements is mostly between 0.1 and 0.3, although with considerable scatter owing to many possible sources for system-specific natural variability and for operational errors^81^. Here, a value of Γ specifically representative for the case of Lake Garda was evaluated by equating Eqs. (21) and (22)^82,83^, which was then used to calculate *K*_*Osborn*_. Using *ε*_*S*_ in Eq. (21) and *χ*_*T*_ in Eq. (22), a median value of Γ = 0.28 was found, by safely excluding segments with *ε*_*S*_ likely affected by shear probes’ noise floor and characterized by very weak stratification (i.e., considering only segments with *ε*_*T*_ > 5 × 10^−10^ m^2^ s^−3^ and *N*^2^ > 5 × 10^−6^ s^−2^). While for the sake of completeness we provide this first order quantification of Γ, we note that there is increasing concern that the mixing efficiency Γ is not always constant, but dependent on the characteristics and evolution of a turbulent event, described in terms of the ratios of the length-scales defining the turbulent range (Ozmidov, Thorpe, Kolmogorov length scales) or the Richardson number, among other parameters^84–88^. The range of conditions where a constant value of Γ is applicable and how Γ depends on the different parameters are the object of a vivid debate within the turbulence community^81^. In conclusion, the extensive microstructure dataset collected in Lake Garda contains all the necessary ingredients to explore the range of applicability of the length-scale dissipation method as well as better constrain the variability of turbulent mixing in a broad range of stratification and energetic conditions, opening new possibilities for future studies and for the reanalysis of existing datasets.

## Data Availability

The scripts for shear spectrum integration and maximum likelihood estimation (MLE) fitting are available together with the dataset (except for some functions of the ODAS libraries released by Rockland Scientific International Inc., to which we refer the interested reader).

## Online-only Table

**Online-only Table 1:** List of the variables stored in the database, with their description and storage group (B for BINNED, S for SLOW, F for FAST, M for METEO).

Variable name (symbol)	Unit of measure	Description	Storage group			
			B	S	F	M
date	—	Date in format yyyy-mm-dd	✓	✓	✓	✓
time	—	UTC time in format HH:MM:SS	✓	✓	✓	✓
filename	—	Name of the raw .p file containing the analyzed profile	✓	✓	✓	
profID	—	Profile ID	✓	✓	✓	
direction	—	Profiling direction: downward or upward	✓	✓	✓	
pressure	dbar	Water pressure	✓	✓	✓	
depth	m	Water depth	✓	✓		
speed (W)	m s^−1^	Vertical profiling speed	✓			
temperature (T)	°C	Water temperature measured by the precise CT	✓	✓		
conductivity (C)	μS cm^−1^	Water conductivity measured by the precise CT	✓	✓		
salinity (Sal)	mg l^−1^	Water salinity, equation (18)	✓	✓		
density (ρ)	kg m^−3^	Water density	✓	✓		
N2 (N^2^)	s^−2^	Background Brunt-Väisälä frequency squared, equation (20)	✓			
LT (L_T_)	m	Thorpe scale from density overturns, equation (19)	✓			
avggradT	°C m^−1^	Segment-averaged temperature gradient based on the data from the precise CT	✓			
maxgradT	°C m^−1^	Local maximum temperature gradient within a segment based on the data from the precise CT	✓			
avggrad*sensor* (<∂T/∂z>)	°C m^−1^	Segment-averaged temperature gradient based on the data from *sensors* T1 and T2 used in equation (22)	✓			
chlorophyll	ppb	Chlorophyll-a measured by the FT sensor	✓		✓	
turbidity	FTU	Turbidity measured by the FT sensor	✓		✓	
fast_*sensor*	s^−1^, °C	Shear and temperature time series for *sensors* S1 and S2, and T1 and T2, respectively			✓	
grad_*sensor*	°C m^−1^	High frequency temperature gradient for *sensors* T1 and T2			✓	
A_x, A_y	counts	High frequency vibrations from the pair of piezo-accelerometers			✓	
Incl_x, Incl_y	°	Instrument pitch and roll from inclinometer		✓		
kB_*sensor* (k_B_)	cpm	Batchelor wavenumber based on ɛ from *sensors* S1, S2, T1, and T2	✓			
eps_*sensor* (ɛ_S_, ɛ_T_)	m^2^ s^−3^	TKE dissipation rate for *sensors* S1, S2, T1, and T2	✓			
Xi_*sensor* (χ_T_)	°C^2^ s^−1^	Temperature variance dissipation rate for *sensors* T1 and T2 (MLE spectral fitting)	✓			
Xi_S*sensor* (χ_ST_)	°C^2^ s^−1^	Temperature variance dissipation rate for *sensors* T1 and T2 (using k_B_ from shear probes)	✓			
MAD_sensor	—	MAD between measured and reference spectra for *sensors* S1, S2, T1, and T2	✓			
MAD_S*sensor*	—	MAD between measured and reference temperature gradient spectra for *sensors* T1 and T2, using k_B_ from shear probes	✓			
MADc	—	Rejection threshold value of MAD	✓			
LR_*sensor* (LR)	—	Likelihood ratio between theoretical and power-law MLE fitting for *sensors*T1 and T2, equation (16)	✓			
kL_*sensor* (k_L_)	cpm	Lower integration wavenumber for *sensors* S1, S2, T1, and T2	✓			
kU_*sensor* (k_U_)	cpm	Upper integration wavenumber for *sensors* S1, S2, T1, and T2	✓			
krange_*sensor*	cpm	Log_10_ of the wavenumber integration range for *sensors* T1, and T2	✓			
kpeak_*sensor* (k_P_)	cpm	Wavenumber corresponding to the peak of the fitted Kraichnan spectrum for *sensors*T1 and T2	✓			
flag_*sensor*	—	Acceptance flag for ɛ and χ estimates for *sensors* S1, S2, T1, and T2 based on the quality metrics: 0=accepted, 1=rejected	✓			
flag_S*sensor*	—	Acceptance flag for χ_ST_ estimates for *sensors* T1 and T2 based on the quality metrics: 0=accepted, 1=rejected	✓			
LO*_sensor* (L_O_)	m	Ozmidov length scale based on ɛ for sensors S1, S2, equation (23)	✓			
KOsborn_*sensor* (K_Osborn_)	m^2^ s^−1^	Diapycnal diffusivity from ɛ for *sensors* S1, S2, T1, and T2, equation (21)	✓			
KOsbornCox_*sensor* (K_Osborn-Cox_)	m^2^ s^−1^	Diapycnal diffusivity from χ for *sensors* T1, and T2, equation (22)	✓			
KOsbornCox_S*sensor* (K_Osborn-Cox_)	m^2^ s^−1^	Diapycnal diffusivity from χ_ST_ for *sensors* T1, and T2, equation (22) (using k_B_ from shear probes)	✓			
AirTemperature	°C	Air temperature from the meteorological station				✓
RelHumidity	%	Relative humidity from the meteorological station				✓
DewPoint	°C	Dew point from the meteorological station				✓
Pressure	mbar	Atmospheric pressure from the meteorological station				✓
WindSpeed	m s^−1^	Wind speed from the meteorological station				✓
SolarRadiation	W m^−2^	Solar radiation from the meteorological station				✓

In the first column, the expression *sensor* should be replaced with S1, S2, T1, and/or T2 as specified in the description (third column).
